# Microchimerism in multiple sclerosis: The association between sex of offspring and MRI features in women with multiple sclerosis

**DOI:** 10.3389/fnins.2023.1091955

**Published:** 2023-02-07

**Authors:** Alessia Bianchi, Maria Aprile, Giuseppe Schirò, Claudia Gasparro, Salvatore Iacono, Michele Andolina, Maurizio Marrale, Irene Gattuso, Giuseppe La Tona, Massimo Midiri, Cesare Gagliardo, Giuseppe Salemi, Paolo Ragonese

**Affiliations:** ^1^Department of Biomedicine, Neuroscience and Advanced Diagnostics, University of Palermo, Palermo, Italy; ^2^Department of Neuroinflammation, University College London, London, United Kingdom; ^3^Department of Physics and Chemistry, University of Palermo, Palermo, Italy; ^4^Neurology Unit, IRCCS San Raffaele Scientific Institute, Milan, Italy

**Keywords:** sex of offspring, pregnancy, microchimerism, sexual chromosomes, multiple sclerosis, magnetic resonance imaging, regional volumes

## Abstract

**Aims:**

During pregnancy, fetal cells can migrate to the mother *via* blood circulation. A percentage of these cells survive in maternal tissues for decades generating a population of fetal microchimeric cells (fMCs), whose biological role is unclear. The aim of this study was to investigate the association between the sex of offspring, an indirect marker of fMCs, and magnetic resonance imaging (MRI) features in women with multiple sclerosis (MS).

**Methods:**

We recruited 26 nulliparous MS patients (NPp), 20 patients with at least one male son (XYp), and 8 patients with only daughters (XXp). Each patient underwent brain MR scan to acquire 3D-T2w FLAIR FatSat and 3D-T1w FSPGR/TFE. Lesion Segmentation Tool (LST) and FreeSurfer were used to obtain quantitative data from MRI. Additional data were collected using medical records. Multiple regression models were applied to evaluate the association between sex of offspring and MS data.

**Results:**

Comparing NPp and XXp, we found that NPp had larger 4th ventricle volume (2.02 ± 0.59 vs. 1.70 ± 0.41; *p* = 0.022), smaller left entorhinal volume (0.55 ± 0.17 vs. 0.68 ± 0.25; *p* = 0.028), and lower thickness in the following cortical areas: left paracentral (2.34 ± 0.16 vs. 2.39 ± 0.17; *p* = 0.043), left precuneus (2.27 ± 0.11 vs. 2.34 ± 0.16; *p* = 0.046), right lateral occipital (2.14 ± 0.11 vs. 2.25 ± 0.08; *p* = 0.006). NPp also had lower thickness in left paracentral cortex (2.34 ± 0.16 vs. 2.46 ± 0.17; *p* = 0.004), left precalcarine cortex (1.64 ± 0.14 vs. 1.72 ± 0.12; *p* = 0.041), and right paracentral cortex (2.34 ± 0.17 vs. 2.42 ± 0.14; *p* = 0.015) when compared to XYp. Comparing XYp and XXp, we found that XYp had higher thickness in left cuneus (1.80 ± 0.14 vs. 1.93 ± 0.10; *p* = 0.042) and left pericalcarine areas (1.59 ± 0.19 vs. 1.72 ± 0.12; *p* = 0.032) and lower thickness in right lateral occipital cortex (2.25 ± 0.08 vs. 2.18 ± 0.13; *p* = 0.027).

**Discussion:**

Our findings suggested an association between the sex of offspring and brain atrophy. Considering the sex of offspring as an indirect marker of fMCs, we speculated that fMCs could accumulate in different brain areas modulating MS neuropathological processes.

## 1. Introduction

Multiple Sclerosis (MS) is a chronic, disabling, autoimmune disorder of the central nervous system (CNS) characterized by both inflammatory and degenerative processes which result in demyelination and axonal damage. Immunological, histopathological, genetic, and therapeutic evidence suggests the autoimmune origin of MS, nonetheless the etiopathogenetic mechanism which triggers the autoimmune reaction and sustains the neurodegeneration has still to be uncovered.

The incidence and prevalence of the disease among women is at least twice the figures for men, with a ratio of women to men that varies throughout geographical areas (The Multiple Sclerosis International Federation [Msif]., 2020). However, the causes behind the greater propensity of women to develop this chronic autoimmune disease during their reproductive years remain unclear and the hormonal differences do not completely explain this phenomenon (Dunn et al., 2015; Kronzer et al., 2020).

Over the last 40 years, several authors have confirmed the existence of persisting fetal cells (fetal microchimeric cells, fMCs) in maternal blood and other tissues, demonstrating that pregnancy may establish a long-term, low-grade chimeric state in the women (Bianchi et al., 1996; O’Donoghue, 2011; Nelson, 2012). Living fMCs transfer from the fetal blood into the maternal circulation during the pregnancy, persist for decades, and determine a microchimeric condition in the mother (Miech, 2010; Nelson, 2012). The effect of fetal microchimerism in long-term maternal health is still controversial (Nelson, 2012; Seppanen et al., 2013; Boddy et al., 2015) and a possible role of fMCs in the autoimmune diseases has been hypothesized by some authors (Boddy et al., 2015; Stevens, 2016). Indeed, several studies suggested that, in the maternal blood, progenitor cells of the fetal immune system are capable of self-renewal, proliferation, differentiation, and activation, resulting in the production of paracrine and autocrine inflammatory cytokines and chemokines that could be involved in autoimmune diseases (Miech, 2010; Pritchard et al., 2012; Seppanen et al., 2013). On the other hand, some authors demonstrated that fMCs may play a role in maternal wound healing (Boddy et al., 2015) and suggested that the murine feto-maternal microchimerism model could be considered a non-invasive cell transplantation model, as it mimics an allogenic transplantation setting (Zeng et al., 2010).

The available data concerning the role of the microchimerism in MS are still limited. Pregnancy is considered to be a protective factor in MS, an effect likely due to the modulation of the immune system and to possible changes in the brain during pregnancy (McCombe and Greer, 2013). However, the effects of fetal microchimerism have not yet been defined and some authors reported a higher rate of microchimerism in affected women when compared with unaffected women (Willer et al., 2006; Bloch et al., 2011; Jafarinia et al., 2020).

The aim of our retrospective, observational study was to investigate the impact of microchimerism on the neurodegenerative and inflammatory processes that characterize the disease evaluating the magnetic resonance (MR) patterns of MS female patients without a history of pregnancy, with at least a male son, and with only female daughters.

In a Supplementary material, we investigate the role of pregnancy in modulating MS features.

## 2. Materials and methods

Fifty-four consecutive patients attending the Center for the Diagnosis and Treatment of Multiple Sclerosis and Demyelinating Disorders of the University Hospital “Paolo Giaccone” of Palermo, Italy and fulfilling the diagnosis of MS according to the revised McDonald criteria (Polman et al., 2011; Thompson et al., 2018) were retrospectively enrolled in the study. Patients were categorized according to their obstetric history in three groups: (1) nulliparous subjects supposedly without fMCs (NPp), (2) subjects with only female daughters, who were supposed to carry only XX fMCs (XXp), and (3) subjects with at least a male son, who were supposedly carriers of XY fMCs (XYp). At recruitment time, 26 patients were nulliparous, 8 had only female daughters, and 20 had at least a male son. As some patients have had a pregnancy in-between onset and recruitment, we also collected data on patients’ pregnancy status at disease onset and diagnosis: we found that, at the first episode, 36 patients were nulliparous, 7 had only female daughters, and 11 had at least a male son. None of the patients had changed status between onset and diagnosis.

Each patient had undergone a 1.5 T magnetic resonance (MR) brain scan at the Neuroradiology Unit of the University Hospital “Paolo Giaccone” of Palermo. The following MR scanners were used: Signa HDxt—GE Medical System, Milwaukee, WI, USA and Achieva—Philips Medical Systems, Cleveland, OH, USA; both systems were equipped with an 8-channel phased-array head coil. The imaging protocol included three-dimensional (3D) T2-weighted (T2w) fluid attenuated inversion recovery (FLAIR) with fat saturation (Fat Sat) and 3D T1-weighted (T1w) fast spoiled gradient echo (FSPGR) pulse sequences or 3D Turbo Field Echo (TFE) pulse sequences on the GE and on the Philips scanner, respectively (technical parameters reported in Table 1). Lesion load volume was segmented from 3D T2w FLAIR sequences by the lesion growth algorithm (Schmidt et al., 2012) as implemented in the Lesion Segmentation Tool (LST)^1^ for SPM. 3D T1w pulse sequences were analyzed to obtain data on whole-brain and regional volumes using FreeSurfer software suite.^2^ MR scanner was included as a categorial covariate in the statistical analysis. LST toolbox and FreeSurfer software were used to obtain quantitative data on white matter, cortical, and subcortical areas. We applied the Aseg, Aparc, Wmparc Free Surfer Atlases to obtain information on regional volumes.

**TABLE 1:** MRI acquisition protocol technical details.

MR pulse sequences	Slice thickness (mm)	TR (ms)	TE (ms)	IT (ms)	Matrix	NEX	FOV (cm)
3D T1w FSPGR (GE)	1	12.4	5.2	450	256 × 256	1	25.6 × 25.6
3D T2w FLAIR fat sat (GE)	1.2	6000	126	1860	224 × 224	1	26.0 × 26.0
3D T1w TFE (Philips)	1	7.4	3.4	–	256 × 256	1	25.6 × 25.6
3D T2w FLAIR fat sat (Philips)	1.12	4800	281	1650	228 × 225	1	26.0 × 26.0

Medical records and questionnaires were used to collect (a) demographic information, (b) prevalence of comorbidity, including every type of comorbidity and specifically other autoimmune (AI) comorbidity, (c) family history for MS or other AI diseases, (d) clinical data at onset, (e) clinical, neuroradiological, and paraclinical data at diagnosis, and (f) clinical features at recruitment.

The study was conducted in accordance with the International Conference on Harmonization guidelines for Good Clinical Practice and the Declaration of Helsinki. All patients gave informed consent upon data collection and analysis.

### 2.1. Statistical analysis

Statistical analysis was performed using STATA software version 17.0 (StataCorp LLC 2021). Demographic, neuroradiological, clinical, and paraclinical characteristics of patients were summarized through counts and percentages for categorical variables. Quantitative variables were reported as mean ± standard deviation (SD) and median and interquartile range (IQR) when the variable distribution was asymmetric.

The analysis of neuroradiological data was performed considering the pregnancy status at the MRI (NPp vs. XXp vs. XYp at recruitment). We built a linear regression model with adjustments for age, disease duration, and MRI scan to evaluate the relationship between the sex of offspring and disease features at recruitment. Additional adjustments for number of previous DMTs and number of previous pregnancies were performed where applicable.

Data at disease onset and diagnosis were analyzed according to the pregnancy status of patients before disease onset (NPp vs. XXp vs. XYp at onset). A linear regression model with adjustments for age at onset were applied to evaluate the association between the sex of offspring and disease features at onset and diagnosis.

## 3. Results

### 3.1. Participant characteristics

Fifty-four consecutive patients were recruited in the study. The NPp had a mean age of 34.7 ± 8.8 years and were younger than XXp (43.8 ± 12.6 years; coeff = 9.096; *p*-value = 0.014; 95% CI = 1.897 to 16.295) and XYp (40.4 ± 7.2 years; coeff = 5.696; *p*-value 0.036; 95% CI = 0.400 to 10.992). We did not find any difference comparing the prevalence of comorbidity and family history for MS or other autoimmune diseases between the three groups (Table 2). Comparing the obstetric history of patients with at least a child, we found that the mean number of pregnancies was non-significantly lower in XXp compared to XYp (1.88 ± 1.46 vs. 2.75 ± 1.29; *p*-value > 0.05), while the time-gap between pregnancy and onset was similar in the two groups (48.7 ± 61.0 months vs. 51.6 ± 48.2; *p*-value > 0.05) (Table 2).

**TABLE 2:** Demographic characteristics at recruitment.

Demographic characteristics	NPp (*n* = 26)	XXp (*n* = 8)	XYp (*n* = 20)	NPp vs. XXp *P*-value (CI)	NPp vs. XYp *P*-value (CI)	XXp vs. XYp *P*-value (CI)
Age, mean ± sd	34.7 ± 8.8	43.8 ± 12.6	40.4 ± 7.2	0.014 (1.897–16.295)	**0.036 (0.400–10.992)**	0.374 (−11.121–4.321)
Comorbidity, prevalence (%)	11/26 (42.31%)	7/8 (87.50%)	13/20 (65.00%)	0.063 (−0.022–0.817)	0.241 (−0.125–0.487)	0.208 (−0.678–0.156)
Others AI comorbidity, prevalence (%)	0/26 (0.00%)	2/8 (25.00%)	3/20 (15.00%)	0.124 (−0.053–0.431)	0.222 (−0.068–0.286)	0.738 (−0.411–0.295)
Family history for MS, prevalence (%)	2/25 (8.00%)	1/8 (12.50%)	3/20 (15.00%)	0.734 (−0.220–0.310)	0.475 (−0.126–0.266)	0.870 (−0.287–0.337)
Family history for non-MS AI diseases, prevalence (%)	3/25 (12.00%)	2/8 (25.00%)	3/20 (15.00%)	0.386 (−0.168–0.428)	0.786 (−0.190–0.250)	0.550 (−0.439–0.239)
Pregnancy (number), mean ± sd	–	1.88 ± 1.46	2.75 ± 1.29	–	–	0.130 (−0.277–2.027)
Total months on pregnancy, mean ± sd	–	15.00 ± 7.35	19.25 ± 7.35	–	–	0.062 (−0.304–11.357)

Bold values represent statistically significant *p*-value.

### 3.2. Differences in clinical features at onset, diagnosis, and last clinical follow-up between the three groups

We also collected clinical, neuroradiological and paraclinical data at onset, diagnosis, and last clinical follow-up (Tables 3, 4). At onset, the NPp were younger than XYp (23.8 ± 7.5 years vs. 34.9 ± 6.8 years; coeff = 11.076; *p*-value < 0.001; 95% CI = 5.816 to 16.335) (Table 3). Comparing these two groups, we also found that NPp had a less frequent pyramidal involvement at onset [6/36 (16.67%) vs. 5/11 (45.45%); coeff = 0.288; *p*-value = 0.047; 95% CI = 0.004 to 0.572] and a more frequent sensory involvement [20/36 (55.56%) vs. 2/11 (18.18%); coeff = −0.374; *p*-value = 0.029; 95% CI = −0.709 to −0.039] (Table 3). At last follow-up visit, we found that NPp were younger (34.7 ± 8.8 years) than XXp (43.8 ± 12.6 years; coeff = 9.096; *p*-value = 0.014; 95% CI = 1.897 to 16.295) and XYp (40.4 ± 7.2 years; coeff = 5.696; *p*-value = 0.036; 95% CI = 0.400 to 10.992) and that the percentage of patients with an Expanded Disability Status Scale (EDSS) score ≥ 3.0 was higher in NPp when compared with XYp [1/26 (38.46%) vs. 6/19 (31.58%); coeff = −0.277; *p*-value = 0.031; 95% CI = −0.527 to −0.026] (Table 4).

**TABLE 3:** Comparison of MS features at onset and diagnosis.

MS features	NPp (*n* = 36)	XXp (*n* = 7)	XYp (*n* = 11)	NPp vs. XXp *P*-value (CI)	NPp vs. XYp *P*-value (CI)	XXp vs. XYp *P*-value (CI)
**MS onset**						
Age, mean ± sd	23.8 ± 7.5	28.7 ± 9.2	34.9 ± 6.8	0.126 (−1.425–11.187)	**< 0.001 (5.816–16.335)**	0.120 (−1.804–14.193)
Polysymptomatic onset, prevalence (%)	10/36 (27.78%)	2/7 (28.57%)	2/11 (18.18%)	0.966 (−0.365–0.380)	0.538 (−0.407–0.215)	0.630 (−0.552–0.345)
Pyramidal symptoms, prevalence (%)	6/36 (16.67%)	1/7 (14.29%)	5/11 (45.45%)	0.889 (−0.364–0.316)	**0.047 (0.004–0.572)**	0.192 (−0.173–0.797)
Visual symptoms, prevalence (%)	11/36 (30.56%)	3/7 (42.86%)	5/11 (45.45%)	0.543 (−0.281–0.527)	0.378 (−0.188–0.486)	0.920 (−0.514–0.566)
Sensory symptoms, prevalence (%)	20/36 (55.56%)	2/7 (28.57%)	2/11 (18.18%)	0.183 (−0.671–0.132)	**0.029 (**−**0.709**–−**0.039)**	0.630 (−0.552–0.345)
Cerebellar symptoms, prevalence (%)	3/36 (8.33%)	0/7 (0.00%)	1/11 (9.09%)	0.455 (−0.305–0.139)	0.935 (−0.178–0.193)	0.442 (−0.153–0.335)
Brainstem symptoms, prevalence (%)	9/36 (25.00%)	3/7 (42.86%)	2/11 (18.18%)	0.336 (−0.190–0.548)	0.658 (−0.376–0.240)	0.281 (−0.716–0.222)
Sphincter symptoms, prevalence (%)	1/36 (2.78%)	1/7 (14.29%)	1/11 (9.09%)	0.235 (−0.077–0.307)	0.433 (−0.097–0.223)	0.751 (−0.392–0.289)
Progression at onset, prevalence (%)	1/36 (2.78%)	0/7 (0.00%)	1/11 (9.09%)	0.728 (−0.187–0.131)	0.345 (−0.070–0.196)	0.442 (−0.153–0.335)
Relapses within the first 3 years from onset, mean ± sd	3.28 ± 2.31	4.29 ± 1.70	2.64 ± 2.11	0.275 (−0.825–2.841)	0.404 (−2.170–0.887)	0.102 (−3.667–0.368)
Time-gap from delivery to onset (months), mean ± sd	–	48.71 ± 61.00	51.55 ± 48.20	–	–	0.725 (−68.133–48.283)
**MS diagnosis**						
Time-gap onset-diagnosis (months), mean ± sd	33.5 ± 86.5	83.7 ± 101.1	27.2 ± 41.9	0.143 (−17.538–118.022)	0.824 (−62.819–50.2381)	0.115 (−128.487–15.422)
EDSS, median (range)	1.5 (0.0–6.0)	3.5 (1.5–4.0)	2.8 (0.0–6.0)	0.537 (−1.164–2.200)	0.447 (−1.744–0.784)	0.175 (−2.835–0.583)
Positive OCBs, prevalence (%)	21/27 (77.78%)	5/6 (83.33%)	5/9 (55.56%)	0.834 (−0.410–0.506)	0.274 (−0.653–0.190)	0.125 (−0.786–0.108)

Bold values represent statistically significant *p*-value.

**TABLE 4:** Comparison of MS features at last clinical follow-up.

MS features	NPp (*n* = 26)	XXp (*n* = 8)	XYp (*n* = 20)	NPp vs. XXp *P*-value (CI)	NPp vs. XYp *P*-value (CI)	XXp vs. XYp *P*-value (CI)
Age, mean ± sd	34.7 ± 8.8	43.8 ± 12.6	40.4 ± 7.2	**0.014 (1.897–16.295)**	**0.036 (0.400–10.992)**	0.374 (−11.121–4.321)
Disease duration (years), mean ± sd	9.4 ± 9.5	16.2 ± 12.4	10.4 ± 7.6	0.080 (−0.841–14.322)	0.730 (−4.612–6.543)	0.143 (−13.637–2.087)
Progression, prevalence (%)	1/26 (3.85%)	0/8 (0.00%)	2/20 (10.00%)	0.350 (−0.293–0.106)	0.671 (−0.115–0.177)	0.354 (−0.132–0.354)
EDSS at follow-up, median (range)	1.5 (0.0–6.5)	4.3 (0.0–7.0)	2.3 (1.0–6.5)	0.829 (−1.254–1.559)	0.245 (−1.630–0.425)	0.252 (−2.557–0.702)
Patients with EDSS ≥ 3.0, prevalence (%)	10/26 (38.46%)	5/8 (62.50%)	6/19 (31.58%)	0.669 (−0.409–0.265)	**0.031 (**−**0.527–−0.026)**	0.201 (−0.643–0.143)
Time from onset to EDSS 3.0 (months), mean ± sd	123.3 ± 151.8	159.8 ± 123.9	48.0 ± 40.4	0.598 (−106.238–179.238)	0.255 (−209.875–59.275)	0.065 (−232.231–8.631)
Annual relapse rate, mean ± sd	0.80 ± 0.45	0.60 ± 0.47	0.76 ± 0.47	0.931 (−0.361–0.331)	0.949 (−0.261–0.245)	0.942 (−0.372–0.346)
DMTs (number), mean ± sd	2.22 ± 1.20	2.00 ± 1.31	1.85 ± 1.04	0.566 (−1.265–0.700)	0.473 (−0.978–0.461)	0.961 (−0.937–0.985)
First-line DMTs (number), mean ± sd	1.52 ± 0.79	1.38 ± 0.52	1.35 ± 0.88	0.479 (−0.928–0.442)	0.558 (−0.649–0.355)	0.775 (−0.574–0.765)
Second-line DMTs (number), mean ± sd	0.70 ± 0.82	0.63 ± 0.92	0.50 ± 0.61	0.905 (−0.703–0.624)	0.646 (−0.598–0.374)	0.825 (−0.721–0.577)
DMTs before EDSS 3.0 (number), mean ± sd	1.77 ± 0.75	1.57 ± 1.13	2.14 ± 0.86	0.706 (−0.980–0.670)	0.219 (−0.249–1.050)	0.241 (−0.421–1.564)
DMTs before EDSS 6.0 (number), mean ± sd	2.20 ± 1.23	2.25 ± 1.26	2.00 ± 0.89	0.463 (−0.949–1.988)	0.865 (−1.305–1.110)	0.888 (−0.076–0.066)
EDSS at last pregnancy, median (range)	–	0.0 (0.0–1.0)	0.0 (0.0–2.0)	–	–	0.692 (−0.860–1.274)
Relapses within 6 months post-partum (number), mean ± sd	–	0.43 ± 0.53	0.00 ± 0.00	–	–	0.801 (−0.353–0.453)
Relapses within 3 years post-partum (number), mean ± sd	–	1.57 ± 1.27	1.09 ± 0.94	–	–	0.502 (−0.857–1.707)

Bold values represent statistically significant *p*-value.

### 3.3. Differences in brain MRI measures between the three groups

Main findings for whole-brain and regional gray and white matter volumes are reported in Tables 5, 6. All regional volumes are detailed in Supplementary Tables 1–3.

**TABLE 5:** Radiological data for whole-brain volumes.

Radiological data	NPp (*n* = 26)	XXp (*n* = 8)	XYp (*n* = 20)	NPp vs. XXp *P*-value (CI)	NPp vs. XYp *P*-value (CI)	XXp vs. XYp *P*-value (CI)
White matter lesion (number), mean ± sd	27.68 ± 26.86	18.25 ± 9.25	24.34 ± 30.49	0.509 (−30.621–15.415)	0.581 (−22.216–12.610)	0.814 (−20.908–26.330)
White matter lesion (volume, cm^3^), mean ± sd	14.17 ± 22.50	11.73 ± 9.99	20.12 ± 37.10	0.484 (−31.475–15.134)	0.984 (−17.808–17.452)	0.739 (−24.502–33.993)
Gray matter (volume, cm^3^), mean ± sd	564.47 ± 58.38	533.15 ± 31.51	562.72 ± 46.58	0.597 (−53.853–31.324)	0.633 (−24.517–39.918)	0.248 (−16.982–62.216)
White matter (volume, cm^3^), mean ± sd	380.57 ± 49.34	365.77 ± 48.06	390.70 ± 57.15	0.684 (−55.382–36.657)	0.567 (−24.833–44.795)	0.361 (−26.459–69.634)
Subcortical structures (volume, cm^3^), mean ± sd	48.03 ± 5.95	46.96 ± 5.55	49.33 ± 5.81	0.778 (−4.237–5.628)	0.329 (−1.905–5.558)	0.588 (−3.876–6.670)
Cortical gray matter (volume, cm^3^), mean ± sd	415.40 ± 46.06	389.72 ± 29.31	414.81 ± 38.82	0.587 (−44.167–25.281)	0.552 (−18.457–34.080)	0.224 (−13.772–55.440)

**TABLE 6:** Radiological data for regional volumes.

Regional volumes	NPp (*n* = 26)	XXp (*n* = 8)	XYp (*n* = 20)	NPp vs. XXp *P*-value (CI)	NPp vs. XYp *P*-value (CI)	XXp vs. XYp *P*-value (CI)
**Subcortical volumes (cm^3^, mean ± sd)**						
4th ventricle	2.02 ± 0.59	1.95 ± 0.50	1.70 ± 0.41	0.233 (−0.688–0.172)	**0.022 (−0.708**—**0.057)**	0.545 (−0.490–0.266)
Right pallidum	1.54 ± 0.23	1.63 ± 0.24	1.68 ± 0.22	0.146 (−0.052–0.340)	**0.049 (0.001**–**0.297)**	0.877 (−0.196–0.228)
**Cortical volumes (mm, mean ± sd)**						
Left cuneus	1.84 ± 0.15	1.80 ± 0.14	1.93 ± 0.10	0.684 (−0.135–0.089)	0.057 (−0.003–0.167)	**0.042 (0.005**–**0.225)**
Left paracentral	2.34 ± 0.16	2.39 ± 0.17	2.46 ± 0.17	**0.043 (0.004**–**0.258)**	**0.004 (0.047–0.238)**	0.741 (−0.130–0.180)
Left pericalcarine	1.64 ± 0.14	1.59 ± 0.19	1.72 ± 0.12	0.571 (−0.152–0.085)	**0.041 (0.004–0.184)**	**0.032 (0.014–0.282)**
Left precuneus	2.27 ± 0.11	2.34 ± 0.16	2.34 ± 0.17	**0.046 (0.002–0.234)**	0.079 (−0.009–0.166)	0.599 (−0.187–0.112)
Right lateral occipital	2.14 ± 0.11	2.25 ± 0.08	2.18 ± 0.13	**0.006 (0.041**–**0.234)**	0.409 (−0.043–0.103)	**0.027 (**−**0.207–**−**0.014)**
Right paracentral	2.34 ± 0.17	2.31 ± 0.23	2.42 ± 0.14	0.407 (−0.078–0.189)	**0.015 (0.026–0.228)**	0.245 (−0.066–0.246)
**White matter volumes (cm^3^, mean ± sd)**						
Left entorhinal	0.55 ± 0.17	0.68 ± 0.25	0.59 ± 0.20	**0.028 (0.021–0.354)**	0.318 (−0.063–0.189)	0.158 (−0.336–0.058)

Bold values represent statistically significant *p*-value.

Analyzing the subcortical structures, the 4th ventricle was larger in NPp group when compared with XYp group (2.02 ± 0.59 vs. 1.70 ± 0.41; coeff = −0.382; *p*-value = 0.022; 95% CI = −0.708 to −0.057) and the right globus pallidus was smaller in NPp group (1.54 ± 0.23 vs. 1.68 ± 0.22; coeff = 0.149; *p*-value = 0.049; 95% CI = 0.001 to 0.297).

In the cortical areas, NPp had a lower thickness in the left paracentral cortex when compared with XXp (2.34 ± 0.16 vs. 2.39 ± 0.17; coeff = 0.131; *p*-value = 0.043; 95% CI = 0.004 to 0.258) and XYp (2.34 ± 0.16 vs. 2.46 ± 0.17; coeff = 0.142; *p*-value = 0.004; 95% CI = 0.047 to 0.238). The same group had a lower thickness in comparison to XXp in left precuneus (2.27 ± 0.11 vs. 2.34 ± 0.16; coeff = 0.118; *p*-value = 0.046; 95% CI = 0.002 to 0.234) and right lateral occipital cortex (2.14 ± 0.11 vs. 2.25 ± 0.08; coeff = 0.138; *p*-value = 0.006; 95% CI = 0.041 to 0.234). NPp also had a lower thickness in comparison to XYp in left precalcarine (1.64 ± 0.14 vs. 1.72 ± 0.12; coeff = 0.094; *p*-value = 0.041; 95% CI = 0.004 to 0.184) and right paracentral areas (2.34 ± 0.17 vs. 2.42 ± 0.14; coeff = 0.127; *p*-value = 0.015; 95% CI = 0.026 to 0.228). Comparing the cortical thickness in patients with at least a child, we found that XYp had a higher thickness in the left cuneus (1.80 ± 0.14 vs. 1.93 ± 0.10; coeff = 0.115; *p*-value = 0.042; 95% CI = 0.005 to 0.225) and in the left pericalcarine cortex (1.59 ± 0.19 vs. 1.72 ± 0.12; coeff = 0.148; *p*-value = 0.032; 95% CI = 0.014 to 0.282) and a lower thickness in right lateral occipital cortex (2.25 ± 0.08 vs. 2.18 ± 0.13; coeff = −0.110; *p*-value = 0.027; 95% CI = −0.207 to −0.014).

Finally, the analysis of white matter volumes demonstrated that the left entorhinal volume was lower in NPp (0.55 ± 0.17) when compared with XXp (0.68 ± 0.25; coeff = 0.188; *p*-value = 0.028; 95% CI = 0.021 to 0.354).

## 4. Discussion

The aim of this hypothesis-generating, retrospective study was to investigate the association between the sex of offspring and neuroradiological features in MS female patients. We collected data on obstetric history of patients and categorized them in 3 groups: (1) NPp were supposed not to have fMCs, (2) XXp were supposed to carry only XX fMCs (XXp), and (3) XYp were supposed to be carriers of XY fMCs. The data showed that NPp had lower brain volumes in several cortical areas, as well as in some subcortical and white matter volumes. More specifically, comparing NPp and XXp, we found that the former group had a larger 4th ventricle and smaller right pallidum and left entorhinal volumes. NPp also reported a lower thickness in left paracentral cortex, left precuneus cortex, and right lateral occipital cortex when compared with XXp. A similar trend was observed comparing NPp and XYp: NPp group had a lower thickness in left paracentral cortex, left precalcarine cortex, and right paracentral cortex. Interestingly, at the comparison between XYp and XXp, we observed that the thickness was higher in XYp in the left cuneus cortex and left pericalcarine cortex, while XXp had a higher thickness in the right lateral occipital cortex.

Pregnancy is considered to be protective in MS (McCombe and Greer, 2013), and interestingly, our results suggest that having a pregnancy is associated with a reduction in cortical and white volume atrophy. Indeed, the results showed that patients with at least a son had developed a lower atrophy in several cortical areas, subcortical volumes, and white matter regions. The neurodegeneration of both gray matter and white matter seems to start in the early phases of MS and to progress throughout the disease course. This phenomenon is considered to be associated not only to the demyelination and the secondary axonal loss, but also to other pathological processes, including the oxidative burst activation of microglia and macrophages, the mitochondrial dysfunction, and the subsequent neuronal energy failure. The occurrence of an hypoxic state triggers the Wallerian degeneration, iron accumulation, meningeal inflammation, and astrocytes activation, starting a cascade that result in axonal loss (Frischer et al., 2009; Kawachi and Lassmann, 2017). We hypothesize that fMCs could modulate these mechanisms.

In Liégeois et al. (1977) proposed the term “microchimerism” to describe long-term donor cell survival in a small proportion relative to the host cell numbers. This phenomenon has been demonstrated in a number of clinical settings, including pregnancy, twinning, transplantation, and blood transfusion (Bloch et al., 2011). Living fetal cells transfer from the fetal blood into the maternal circulation during the pregnancy. After the delivery, most of these cells are actively destroyed by the maternal immune system through apoptotic processes (Kolialexi et al., 2004; Pritchard et al., 2012). Nonetheless, a small percentage of fMCs survive, persisting for decades and establishing a long-term, low-grade chimeric state in the human female (Bianchi et al., 1996; O’Donoghue, 2011; Nelson, 2012). The survival fMCs have been reported to be capable of colonize maternal tissues, including the CNS (Kaplan and Land, 2005; Tan et al., 2005; Rijnink et al., 2015), but the effect of fetal microchimerism in long-term maternal health is still controversial (Nelson, 2012; Seppanen et al., 2013; Boddy et al., 2015).

A possible role of fMCs in autoimmune diseases has been hypothesized (Boddy et al., 2015; Stevens, 2016), but the effect of this phenomenon in MS remain undefined and few studies have been conducted to investigate it. Some authors reported a higher rate of microchimerism in affected women when compared with unaffected subjects (Willer et al., 2006; Bloch et al., 2011; Jafarinia et al., 2020). In addition, Snethen et al. (2020) investigated the presence and distribution of maternal microchimeric cells in adult human brain of MS patients, demonstrating that the largest portion of female cells were of neuronal or, in a smaller proportion, immune lineage and that the frequency of the cells was higher in active lesions when compared with normal appearing MS samples and control samples.

In Tan et al. (2005) reported that at least some of the fMCs that spontaneously migrate in the maternal circulation during pregnancy are capable of entering the brain. The same group suggested that, in case of injury, inflammatory signaling pathways can activate fMCs migration to sites of damage (Tan et al., 2005), where they undergo cellular and molecular maturation processes similar to those observed in adult neurogenesis (Zeng et al., 2010). The same group confirmed their hypothesis analyzing mice mothers with experimentally induced Parkinson’s disease and observing an increase in fMCs in the lesioned areas of the brain (Zeng et al., 2010). The authors concluded that feto-maternal microchimerism in the brain represents a model of neural differentiation similar to adult neurogenesis and that fMCs can activate processes of cell maturation and time-dependent integration into the brain, inducing a “regeneration” that could act a role in neurodegenerative diseases (Zeng et al., 2010). Moreover, in Chan et al. (2012) evaluated the role of male fMCs in women brain affected by Alzheimer’s disease (AD). The findings suggested that AD patients had a lower prevalence of male fMCs in the brain and, particularly, in the regions most affected by the disease and that AD was significantly associated with lower fMCs prevalence (Chan et al., 2012).

The data reported in this study showed that women with at least a previous pregnancy had higher regional brain volumes and thickness. Several pregnancy-associated changes, including circulating hormones and epigenetic changes, have been previously suggested as protective variables in pregnancy and it is possible to speculate that the same biological mechanisms could influence the neuroradiological outcomes (McCombe and Greer, 2013; Graves, 2020). However, the differences found in comparing (a) NPp vs. XXp and (b) NPp vs. XYp and the differences identified comparing (c) XXp vs. XYp could not be related to changes occurring in both male and female pregnancies, such as hormonal and epigenetic modifications. On contrary, these differences should be due to a factor that varies from male and female pregnancies. We hypothesized that, in a multifactorial background and likely interacting with other pregnancy-relate factors, microchimerism could be a good candidate for the role. Indeed, previous studies have demonstrated the role of sexual chromosome in modulating the immune system and authors agreed on the disease-promoting effect of XX chromosome compared to XY complement (Smith-Bouvier et al., 2008). Evidences suggest that the female immune response would be more robust that the male one (Nicot, 2009; Voskuhl et al., 2018), while XY genotype could be associated to neurodegeneration and a subsequent more severe disability (Smith-Bouvier et al., 2008; Voskuhl et al., 2018). The sexual chromosomes in fMCs are a distinctive factor between male and female pregnancies, while other pregnancy-associated changes are thought to be similar in male and female offspring. Therefore, the results of this study were in line with the protective effect of pregnancy previously described (McCombe and Greer, 2013), but making a step forward, they also supported the speculation that the differences observed comparing XXp and XYp to NPp and comparing XXp and XYp could be the results of XX and XY fMCs. Indeed, we hypothesized that XY and XX fMCs could act differently in the inflammatory and neurodegenerative processes, explaining the differences observed in our population between patients with only daughters and patients with at least a male son. Pregnancy is protective in MS and this effect seems to be associated to a modulation of the immune system (McCombe and Greer, 2013): the differences observed comparing XYp and XXp suggest that MCs can have an effect in this modulation, supporting the hypothesis of a possible role of microchimerism in MS.

Proceeding from the available literature and the findings reported in this study, we speculated that these fMCs could act a neuroprotective role, homing in gray matter and white matter areas, activating processes of neurogenesis and circuit integration, and modulating the neurodegeneration associated to MS. It is worth to note that most of the differences were observed in cortical areas: in Masseyeff et al. (1983) speculated that fMCs preferentially homed to maternal lymphoid organs where they possessed suppressor characteristics and it is now clear that the intrathecal inflammation associated to the clinical and pathological progression in MS is characterized by the accumulation of B-cells in tertiary lymphoid-like structures within the inflamed meninges (Monaco et al., 2020). In line with these previous findings, we hypothesize that the higher effect of fMCs in cortical areas could be related to their migration in the lymphoid-like structures, where they might also modulate the meningeal inflammation. Tissue-based analysis demonstrated that fMCs are not homogeneously distributed in maternal organs, including the brain. Walczak et al. (2004) and Tan et al. (2005) found that in mice fMCs preferentially colonize the region of the olfactory bulb, an area that has been reported to support survival and limited proliferation, migration, and immunocytochemical differentiation of progenitor cells. Moreover, the authors observed that fMCs occurred in clusters and hypothesized that these cells could colonize facilitating niches, where they can proliferate and survive and, from where, they can migrate attracted by signaling molecules (Tan et al., 2005). In line with these findings, we hypothesized that the localized differences detected in our study could be related to the inhomogeneous distribution of fMCs in maternal gray and white matters.

The retrospective selection of patients and the subsequently lack of blood samples at MRI acquisition for the direct research of fMCs constitute the main limitations of our study. To overcome this potential bias, data were collected from official medical records and information about pregnancies and abortions were obtained directly from the patients. Therefore, the available literature on acquisition of microchimeric cells during pregnancy and the high accuracy in data collection support our classification of patients. A second limitation is linked to the unbalanced numerosity between the three groups, which could have reduced the statistical power in some analysis and covered additional differences. Moving from these hypothesis-generating results, we designed a prospective, longitudinal study on a larger population of patients, which is still in progress.

In conclusion, to our knowledge, this is the first study investigating the neuroradiological differences in MS patients according to the sex of offspring as an indirect marker of fMCs. The main limitation of the study is related to the lack of dosage of fMCs in patient blood sample. Nonetheless, our findings suggested that having a male or a female son is associated to different MRI features in some areas. Further studies are required to determine whether this phenomenon could be related to feto-maternal microchimerism and the role of fMCs in neurodegenerative diseases.

## Data availability statement

The raw data supporting the conclusions of this article will be made available by the authors, without undue reservation.

## Ethics statement

Ethical approval was not provided for this study on human participants because data for this project were collected from clinical practice. The patients/participants provided their written informed consent to participate in this study.

## Author contributions

AB and PR have made a substantial contribution to the concept, design, and conduction of the study and drafted the article or revised it critically for important intellectual content. AB have contributed to the statistical analysis of the data. AB, MaA, GSc, ClG, SI, and MiA have contributed to the data collection for the article. CeG, MMa, GL, MMi, and IG have collected and processed the MRI data for this project. All authors approved the version to be published.

## Footnotes

1
www.statistical-modeling.de/lst.html

2
https://surfer.nmr.mgh.harvard.edu/
