# Supplementary material for: Microchimerism in multiple sclerosis: The association between sex of offspring and MRI features in women with multiple sclerosis
Source: Front Neurosci. 2023 Feb 7;17:1091955. doi: 10.3389/fnins.2023.1091955 (PMC9941336; doi:10.3389/fnins.2023.1091955)
Supplement: Supplementary file 1 [file Data_Sheet_1.PDF]

Supplementary Material.

Supplemental statistical analysis.

To investigate the role of pregnancy in modulating MS features, we categorised patients according to their obstetric history in (1) nulliparous subjects (NPp) and (2) subjects with a previous pregnancy (parous patients, Pp). At recruitment time, 26 patients were nulliparous and 28 had at least a previous pregnancy. As some patients have had a pregnancy in-between onset and recruitment, we also collected data on patients' pregnancy status at disease onset and diagnosis: we found that, at the first episode, 36 patients were nulliparous and 18 had at least a previous pregnancy. None of the patients had changed status between onset and diagnosis.

The analysis of neuroradiological data was performed considering the pregnancy status at the MRI (NPp vs Pp at recruitment). We built a linear regression model with adjustments for age, disease duration, and MRI scan to evaluate the relationship between pregnancy and disease features at recruitment. Additional adjustments for number of previous DMTs and number of previous pregnancies were performed where applicable.

Data at disease onset and diagnosis were analysed according to the pregnancy status of patients before disease onset (NPp vs Pp at onset). A linear regression model with adjustments for age at onset were applied to evaluate the association between pregnancy and disease features at onset and diagnosis.

Supplemental results.

We found that NPp were younger at last clinical follow-up than Pp (41.0 ± 9.2 vs 34.5 ± 8.5; coeff = 6.500, p-value = 0.010, 95%CI = 1.612 to 11.388). At onset, Pp were older than NPp (31.9 ± 8.1

vs 23.7 ± 7.7; coeff = 8.144, p-value = 0.001, 95%CI = 3.722 to 12.566) and the former group also reported higher percentage of patients who recovered completely after the first relapse (18/19 [94.74%] vs 21/30 [70.00%]; coeff = -0.247, p-value = 0.037, 95%CI = -0.479 to -0.016). NPp had bigger 4th ventricle volumes than Pp (2.04 ± 0.58 vs 1.78 ± 0.46; coeff = -0.400, p-value = 0.008; 95%CI = -0.690 to -0.110), while Pp had higher right pallidum volumes (1.65 ± 0.22 vs 1.55 ± 0.24; coeff = 0.137, p-value = 0.048, 95%CI = 0.001 to 0.272). Pp also had higher thickness in left paracentral cortex (2.42 ± 0.18 vs 2.35 ± 0.15; coeff = 0.120, p-value = 0.009, 95%CI = 0.031 to 0.209) and higher volumes in left superior parietal white matter when compared to NPp (10.45 ± 1.26 vs 9.73 ± 0.95; coeff = 0.742, p-value = 0.031, 95%CI = 0.070 to 1.414).

Supplemental discussion.

We found that nulliparous patients were younger at disease onset and had lower percentage of complete recovery after the first relapse. We also found that NPp had bigger 4th ventricle volumes than Pp and lower right pallidum volumes, left paracentral cortex thickness and left superior parietal white matter volumes.

Our findings confirmed the protective role of pregnancy and are in line with a recent study by Nguyen et al. to investigate the association between pregnancy and time to clinically isolated syndrome (CIS) onset. The authors reported that women with previous pregnancies and childbirths had a later disease onset when compared to women who had never been pregnant and those who had never given birth (Nguyen *et al.*, 2020). Nguyen et al. observed a median delayed of 3.3 years in women with previous pregnancies and 3.4 years in women with previous childbirths, while the number of previous pregnancies and childbirth was not associated with a later onset (Nguyen *et*

al., 2020). The group did not propose any causative factors for explaining this association. However, several pregnancy-associated changes, including circulating hormones and epigenetic changes, have been previously suggested as protective variables in pregnancy and it is possible to speculate that the same biological mechanisms could suppress or postpone the first demyelinating episode and improve the outcome after the first relapse (McCombe and Greer, 2013; Graves, 2020). We hypothesised that microchimerism could be one of these modulating factors. Unfortunately, due to the retrospective nature of the study, it was not possible to investigate the presence of fMCs at onset, so it was not possible to determine whether our findings were related to fMCs or to other pregnancy-related factors.

We also found that NPp had lower brain volumes in right pallidum, left paracentral cortex and left superior parietal white matter volumes, along with a larger 4th ventricle, an indirect marker of brain atrophy. In a recent publication, Lorefice and al. reported no relationships between pregnancy and whole-brain MRI measurements (Lorefice *et al.*, 2022) and our findings confirmed the results. However, the detection of regional differences between the two groups let speculate that an inhomogeneously distributed, pregnancy-associated factor could act as a protective variable in pregnancy. We hypothesised that microchimerism could play this role, nevertheless further studies are necessary to investigate the hypothesis.

Supplementary tables.

Supplementary Table 1. Subcortical volumes (cm^3, mean ± sd)

Subcortical volumes	NLp (n=26)	XXp (n=8)	XYp (n=20)	NLp vs XXp p-value (CI)	NLp vs XYp p-value (CI)	XXp vs XYp p-value (CI)
Left lateral ventricle	10.84 ± 9.02	13.31 ± 9.73	9.62 ± 5.59	0.302 (-8.197 – 2.601)	0.095 (-7.544 – 0.625)	0.502 (-8.402 – 4.249)
Left inferior lateral ventricle	0.42 ± 0.20	0.47 ± 0.21	0.43 ± 0.24	0.971 (-0.177 – 0.183)	0.666 (-0.165 – 0.107)	0.649 (-0.275 – 0.175)
Left cerebellum WM	11.91 ± 1.60	12.18 ± 1.81	12.30 ± 1.92	0.289 (-0.647 – 2.122)	0.469 (-0.668 – 1.428)	0.687 (-1.977 – 1.328)
Left cerebellum cortex	50.07 ± 5.12	47.90 ± 5.59	48.91 ± 3.87	0.587 (-5.167 – 2.961)	0.565 (-3.959 – 2.189)	0.920 (-3.912 – 4.315)
Left thalamus	6.30 ± 0.94	5.88 ± 1.03	6.41 ± 0.91	0.750 (-0.938 – 0.681)	0.423 (-0.366 – 0.859)	0.390 (-0.526 – 1.293)
Left caudate	3.03 ± 0.45	3.06 ± 0.41	3.02 ± 0.44	0.738 (-0.329 – 0.461)	0.944 (-0.309 – 0.288)	0.659 (-0.497 – 0.321)
Left putamen	3.92 ± 0.81	3.64 ± 0.74	4.05 ± 0.62	0.900 (-0.665 – 0.586)	0.326 (-0.240 – 0.707)	0.249 (-0.267 – 0.975)
Left pallidum	1.54 ± 0.25	1.54 ± 0.19	1.65 ± 0.25	0.652 (-0.161 – 0.255)	0.152 (-0.044 – 0.271)	0.381 (-0.123 – 0.309)
3rd ventricle	1.16 ± 0.46	1.44 ± 0.83	1.15 ± 0.50	0.709 (-0.371 – 0.542)	0.389 (-0.494 – 0.196)	0.459 (-0.779 – 0.364)
4th ventricle	2.02 ± 0.59	1.95 ± 0.50	1.70 ± 0.41	0.233 (-0.688 – 0.172)	**0.022 (-0.708 – -0.057)**	0.545 (-0.490 – 0.266)
Brainstem	18.23 ± 2.20	17.85 ± 2.76	19.04 ± 2.23	0.740 (-1.566 – 2.188)	0.191 (-0.483 – 2.357)	0.430 (-1.389 – 3.146)
Left hippocampus	3.49 ± 0.39	3.46 ± 0.36	3.58 ± 0.35	0.872 (-0.302 – 0.354)	0.394 (-0.142 – 0.354)	0.617 (-0.253 – 0.416)
Left amygdala	1.25 ± 0.13	1.24 ± 0.18	1.30 ± 0.27	0.949 (-0.167 – 0.178)	0.659 (-0.102 – 0.159)	0.835 (-0.203 – 0.249)
Left accumbens area	0.47 ± 0.13	0.41 ± 0.13	0.49 ± 0.10	0.883 (-0.106 – 0.091)	0.200 (-0.026 – 0.122)	0.201 (-0.038 – 0.169)
Left ventral DC	3.35 ± 0.43	3.30 ± 0.45	3.45 ± 0.45	0.525 (-0.235 – 0.455)	0.339 (-0.136 – 0.386)	0.771 (-0.356 – 0.474)
Left vessel	0.03 ± 0.03	0.03 ± 0.03	0.04 ± 0.04	0.890 (-0.032 – 0.027)	0.444 (-0.014 – 0.031)	0.290 (-0.015 – 0.048)
Left choroid plexus	0.62 ± 0.39	0.61 ± 0.19	0.53 ± 0.23	0.133 (-0.412 – 0.056)	0.094 (-0.327 – 0.027)	0.645 (-0.248 – 0.157)
Right lateral ventricle	9.87 ± 7.99	11.75 ± 8.27	8.81 ± 5.19	0.273 (-7.537 – 2.180)	0.105 (-6.697 – 0.653)	0.580 (-7.164 – 4.115)
Right inferior lateral ventricle	0.46 ± 0.22	0.48 ± 0.22	0.51 ± 0.32	0.582 (-0.286 – 0.162)	0.915 (-0.160 – 0.178)	0.707 (-0.233 – 0.337)
Right cerebellum WM	11.41 ± 1.59	11.67 ± 1.76	12.10 ± 1.75	0.197 (-0.454 – 2.140)	0.126 (-0.221 – 1.742)	0.947 (-1.564 – 1.466)
Right cerebellum cortex	50.28 ± 5.50	47.83 ± 5.75	49.21 ± 4.03	0.573 (-5.529 – 3.095)	0.720 (-3.847 – 2.677)	0.769 (-3.568 – 4.762)

Right thalamus	5.76 ± 0.86	5.51 ± 0.81	5.99 ± 0.91	0.995 (-0.743 – 0.738)	0.272 (-0.251 – 0.869)	0.399 (-0.481 – 1.159)
Right caudate	3.14 ± 0.47	3.19 ± 0.48	3.12 ± 0.45	0.454 (-0.252 – 0.554)	0.878 (-0.281 – 0.328)	0.479 (-0.567 – 0.275)
Right putamen	4.00 ± 0.77	3.87 ± 0.55	4.07 ± 0.76	0.738 (-0.517 – 0.724)	0.564 (-0.334 – 0.605)	0.795 (-0.572 – 0.737)
Right pallidum	1.54 ± 0.23	1.63 ± 0.24	1.68 ± 0.22	0.146 (-0.052 – 0.340)	**0.049 (0.001 – 0.297)**	0.877 (-0.196 – 0.228)
Right hippocampus	3.60 ± 0.44	3.63 ± 0.36	3.66 ± 0.44	0.625 (-0.283 – 0.466)	0.662 (-0.221 – 0.346)	0.922 (-0.400 – 0.364)
Right amygdala	1.42 ± 0.19	1.42 ± 0.17	1.44 ± 0.29	0.854 (-0.187 – 0.225)	0.718 (-0.128 – 0.184)	0.973 (-0.255 – 0.247)
Right accumbens area	0.44 ± 0.10	0.42 ± 0.09	0.46 ± 0.09	0.646 (-0.057 – 0.091)	0.208 (-0.020 – 0.091)	0.556 (-0.055 – 0.100)
Right ventral DC	3.31 ± 0.44	3.28 ± 0.42	3.43 ± 0.44	0.409 (-0.204 – 0.493)	0.194 (-0.091 – 0.436)	0.712 (-0.318 – 0.457)
Right vessel	0.04 ± 0.04	0.04 ± 0.03	0.04 ± 0.04	0.812 (-0.028 – 0.035)	0.597 (-0.030 – 0.017)	0.763 (-0.039 – 0.029)
Right choroid plexus	0.64 ± 0.26	0.66 ± 0.19	0.59 ± 0.27	0.245 (-0.293 – 0.077)	0.181 (-0.234 – 0.046)	0.803 (-0.263 – 0.206)
Optic chiasm	0.20 ± 0.04	0.22 ± 0.04	0.19 ± 0.04	0.802 (-0.030 – 0.039)	0.066 (-0.051 – 0.002)	0.108 (-0.070 – 0.008)
CC Posterior	0.86 ± 0.26	0.87 ± 0.13	0.80 ± 0.14	0.448 (-0.234 – 0.105)	0.181 (-0.215 – 0.042)	0.217 (-0.199 – 0.048)
CC Middle posterior	0.48 ± 0.16	0.43 ± 0.12	0.42 ± 0.11	0.194 (-0.203 – 0.042)	0.135 (-0.162 – 0.023)	0.789 (-0.121 – 0.093)
CC Central	0.44 ± 0.12	0.39 ± 0.08	0.42 ± 0.11	0.421 (-0.141 – 0.060)	0.745 (-0.088 – 0.064)	0.660 (-0.076 – 0.118)
CC Middle anterior	0.42 ± 0.12	0.34 ± 0.11	0.41 ± 0.13	0.155 (-0.190 – 0.031)	0.734 (-0.098 – 0.069)	0.332 (-0.060 – 0.169)
CC Anterior	0.76 ± 0.15	0.66 ± 0.21	0.78 ± 0.14	0.151 (-0.245 – 0.039)	0.684 (-0.086 – 0.129)	0.211 (-0.056 – 0.240)

Supplementary Table 2. Cortical thickness (mm, mean ± sd)

Cortical thickness	NLp (n=26)	XXp (n=8)	XYp (n=20)	NLp vs XXp p-value (CI)	NLp vs XYp p-value (CI)	XXp vs XYp p-value (CI)
**Left bank of the superior temporal sulcus**	2.42 ± 0.15	2.42 ± 0.16	2.40 ± 0.19	0.282 (-0.062 – 0.208)	0.995 (-0.102 – 0.102)	0.610 (-0.224 – 0.135)
**Left caudal anterior cingulate**	2.57 ± 0.31	2.61 ± 0.33	2.54 ± 0.27	0.927 (-0.272 – 0.248)	0.547 (-0.256 – 0.137)	0.579 (-0.362 – 0.207)
**Left caudal middle frontal**	2.52 ± 0.16	2.49 ± 0.17	2.53 ± 0.13	0.544 (-0.085 – 0.160)	0.454 (-0.058 – 0.127)	0.736 (-0.116 – 0.162)
**Left cuneus**	1.84 ± 0.15	1.80 ± 0.14	1.93 ± 0.10	0.684 (-0.135 – 0.089)	0.057 (-0.003 – 0.167)	**0.042 (0.005 – 0.225)**
**Left entorhinal**	3.19 ± 0.26	3.10 ± 0.31	3.23 ± 0.37	0.968 (-0.276 – 0.265)	0.491 (-0.134 – 0.275)	0.734 (-0.254 – 0.354)
**Left fusiform**	2.66 ± 0.16	2.70 ± 0.25	2.65 ± 0.17	0.357 (-0.085 – 0.230)	0.824 (-0.132 – 0.106)	0.289 (-0.279 – 0.087)
**Left inferior parietal**	2.40 ± 0.10	2.44 ± 0.13	2.41 ± 0.15	0.144 (-0.027 – 0.179)	0.673 (-0.062 – 0.094)	0.289 (-0.193 – 0.061)
**Left inferior temporal**	2.77 ± 0.17	2.74 ± 0.19	2.79 ± 0.21	0.743 (-0.200 – 0.144)	0.856 (-0.118 – 0.142)	0.714 (-0.166 – 0.238)
**Left isthmus cingulate**	2.14 ± 0.22	2.21 ± 0.15	2.11 ± 0.14	0.211 (-0.058 – 0.256)	0.686 (-0.143 – 0.095)	0.119 (-0.245 – 0.030)
**Left lateral occipital**	2.10 ± 0.12	2.17 ± 0.09	2.14 ± 0.14	0.091 (-0.014 – 0.187)	0.494 (-0.050 – 0.103)	0.311 (-0.157 – 0.052)
**Left lateral orbitofrontal**	2.69 ± 0.22	2.77 ± 0.28	2.66 ± 0.23	0.354 (-0.114 – 0.311)	0.798 (-0.181 – 0.140)	0.228 (-0.381 – 0.096)
**Left lingual**	2.02 ± 0.13	1.98 ± 0.09	2.07 ± 0.08	0.993 (-0.090 – 0.089)	0.135 (-0.017 – 0.119)	0.088 (-0.011 – 0.144)
**Left medial orbitofrontal**	2.52 ± 0.17	2.58 ± 0.28	2.50 ± 0.15	0.248 (-0.067 – 0.254)	0.836 (-0.134 – 0.109)	0.222 (-0.307 – 0.075)
**Left middle temporal**	2.79 ± 0.14	2.80 ± 0.17	2.77 ± 0.16	0.210 (-0.044 – 0.193)	0.760 (-0.103 – 0.076)	0.336 (-0.215 – 0.077)
**Left parahippocampal**	2.76 ± 0.25	2.69 ± 0.39	2.80 ± 0.35	0.971 (-0.262 – 0.272)	0.421 (-0.120 – 0.283)	0.847 (-0.287 – 0.346)
**Left paracentral**	2.34 ± 0.16	2.39 ± 0.17	2.46 ± 0.17	**0.043 (0.004 – 0.258)**	**0.004 (0.047 – 0.238)**	0.741 (-0.130 – 0.180)
**Left cprs opercularis**	2.55 ± 0.15	2.57 ± 0.15	2.51 ± 0.16	0.177 (-0.040 – 0.211)	0.542 (-0.124 – 0.066)	0.112 (-0.250 – 0.028)
**Left pars orbitalis**	2.72 ± 0.19	2.72 ± 0.28	2.73 ± 0.24	0.453 (-0.120 – 0.264)	0.663 (-0.114 – 0.177)	0.648 (-0.284 – 0.181)
**Left pars triangularis**	2.44 ± 0.14	2.48 ± 0.21	2.44 ± 0.14	0.059 (-0.004 – 0.217)	0.847 (-0.075 – 0.092)	0.123 (-0.229 – 0.029)
**Left pericalcarine**	1.64 ± 0.14	1.59 ± 0.19	1.72 ± 0.12	0.571 (-0.152 – 0.085)	**0.041 (0.004 – 0.184)**	**0.032 (0.014 – 0.282)**
**Left postcentral**	2.07 ± 0.12	2.03 ± 0.16	2.09 ± 0.10	0.669 (-0.076 – 0.117)	0.141 (-0.019 – 0.128)	0.396 (-0.064 – 0.154)
**Left posterior cingulate**	2.42 ± 0.15	2.50 ± 0.23	2.40 ± 0.16	0.169 (-0.044 – 0.244)	0.662 (-0.132 – 0.085)	0.057 (-0.309 – 0.005)
**Left precentral**	2.50 ± 0.19	2.46 ± 0.18	2.52 ± 0.14	0.460 (-0.084 – 0.182)	0.319 (-0.050 – 0.151)	0.697 (-0.113 – 0.166)

**Left precuneus**	2.27 ± 0.11	2.34 ± 0.16	2.34 ± 0.17	**0.046 (0.002 – 0.234)**	0.079 (-0.009 – 0.166)	0.599 (-0.187 – 0.112)
**Left rostral anterior cingulate**	2.79 ± 0.19	2.76 ± 0.31	2.88 ± 0.34	0.804 (-0.200 – 0.257)	0.140 (-0.044 – 0.302)	0.391 (-0.165 – 0.405)
**Left rostral middle frontal**	2.37 ± 0.14	2.34 ± 0.19	2.41 ± 0.14	0.801 (-0.108 – 0.139)	0.267 (-0.041 – 0.146)	0.631 (-0.112 – 0.180)
**Left superior frontal**	2.66 ± 0.18	2.61 ± 0.21	2.71 ± 0.14	0.564 (-0.099 – 0.180)	0.092 (-0.015 – 0.196)	0.412 (-0.092 – 0.215)
**Left superior parietal**	2.14 ± 0.10	2.13 ± 0.14	2.17 ± 0.14	0.508 (-0.066 – 0.131)	0.489 (-0.049 – 0.100)	0.852 (-0.134 – 0.112)
**Left superior temporal**	2.70 ± 0.16	2.63 ± 0.22	2.68 ± 0.19	0.780 (-0.118 – 0.156)	0.940 (-0.100 – 0.108)	0.898 (-0.180 – 0.159)
**Left supramarginal**	2.52 ± 0.13	2.51 ± 0.17	2.54 ± 0.16	0.258 (-0.050 – 0.184)	0.274 (-0.040 – 0.137)	0.856 (-0.162 – 0.135)
**Left frontal pole**	2.68 ± 0.25	2.70 ± 0.16	2.75 ± 0.20	0.579 (-0.140 – 0.248)	0.285 (-0.068 – 0.226)	0.746 (-0.156 – 0.215)
**Left temporal pole**	3.57 ± 0.33	3.60 ± 0.49	3.52 ± 0.40	0.716 (-0.280 – 0.404)	0.613 (-0.324 – 0.193)	0.449 (-0.567 – 0.260)
**Left transverse temporal**	2.37 ± 0.25	2.30 ± 0.26	2.35 ± 0.17	0.291 (-0.072 – 0.234)	0.410 (-0.068 – 0.163)	0.880 (-0.179 – 0.155)
**Left insula**	3.05 ± 0.17	3.10 ± 0.08	3.00 ± 0.16	0.095 (-0.019 – 0.231)	0.656 (-0.116 – 0.074)	0.133 (-0.237 – 0.033)
**Right bank of the superior temporal sulcus**	2.51 ± 0.17	2.51 ± 0.19	2.51 ± 0.16	0.543 (-0.097 – 0.182)	0.685 (-0.084 – 0.127)	0.604 (-0.198 – 0.118)
**Right caudal anterior cingulate**	2.44 ± 0.28	2.43 ± 0.26	2.49 ± 0.32	0.617 (-0.186 – 0.310)	0.384 (-0.106 – 0.269)	0.648 (-0.227 – 0.358)
**Right caudal middle frontal**	2.51 ± 0.17	2.43 ± 0.15	2.50 ± 0.17	0.934 (-0.117 – 0.127)	0.729 (-0.076 – 0.108)	0.793 (-0.121 – 0.156)
**Right cuneus**	1.90 ± 0.16	1.87 ± 0.12	1.95 ± 0.11	0.922 (-0.126 – 0.114)	0.199 (-0.032 – 0.150)	0.169 (-0.035 – 0.186)
**Right entorhinal**	3.29 ± 0.35	3.11 ± 0.43	3.25 ± 0.34	0.546 (-0.411 – 0.220)	0.860 (-0.260 – 0.218)	0.726 (-0.265 – 0.374)
**Right fusiform**	2.65 ± 0.15	2.68 ± 0.24	2.65 ± 0.14	0.501 (-0.093 – 0.188)	0.833 (-0.118 – 0.095)	0.343 (-0.232 – 0.084)
**Right inferior parietal**	2.44 ± 0.10	2.43 ± 0.15	2.42 ± 0.15	0.401 (-0.056 – 0.136)	0.671 (-0.088 – 0.057)	0.253 (-0.183 – 0.051)
**Right inferior temporal**	2.72 ± 0.14	2.73 ± 0.26	2.80 ± 0.22	0.739 (-0.194 – 0.139)	0.618 (-0.095 – 0.158)	0.676 (-0.174 – 0.264)
**Right isthmus cingulate**	2.19 ± 0.18	2.21 ± 0.18	2.15 ± 0.23	0.489 (-0.109 – 0.224)	0.581 (-0.161 – 0.091)	0.218 (-0.304 – 0.074)
**Right lateral occipital**	2.14 ± 0.11	2.25 ± 0.08	2.18 ± 0.13	**0.006 (0.041 – 0.234)**	0.409 (-0.043 – 0.103)	**0.027 (-0.207 – -0.014)**
**Right lateral orbitofrontal**	2.67 ± 0.24	2.71 ± 0.32	2.66 ± 0.25	0.819 (-0.201 – 0.253)	0.860 (-0.187 – 0.157)	0.602 (-0.331 – 0.197)
**Right lingual**	2.05 ± 0.11	1.98 ± 0.15	2.07 ± 0.12	0.318 (-0.140 – 0.046)	0.944 (-0.073 – 0.068)	0.372 (-0.059 – 0.151)
**Right medial orbitofrontal**	2.57 ± 0.15	2.58 ± 0.28	2.53 ± 0.21	0.820 (-0.152 – 0.192)	0.464 (-0.178 – 0.082)	0.626 (-0.275 – 0.169)
**Right middle temporal**	2.80 ± 0.15	2.84 ± 0.13	2.78 ± 0.19	0.206 (-0.048 – 0.215)	0.519 (-0.132 – 0.067)	0.095 (-0.269 – 0.023)
**Right parahippocampal**	2.69 ± 0.23	2.65 ± 0.30	2.72 ± 0.30	0.989 (-0.232 – 0.235)	0.604 (-0.131 – 0.222)	0.726 (-0.234 – 0.330)

**Right paracentral**	2.34 ± 0.17	2.31 ± 0.23	2.42 ± 0.14	0.407 (-0.078 – 0.189)	**0.015 (0.026 – 0.228)**	0.245 (-0.066 – 0.246)
**Right cprs opercularis**	2.51 ± 0.15	2.48 ± 0.18	2.51 ± 0.20	0.336 (-0.063 – 0.181)	0.710 (-0.075 – 0.110)	0.545 (-0.196 – 0.107)
**Right pars orbitalis**	2.74 ± 0.19	2.71 ± 0.22	2.71 ± 0.19	0.897 (-0.159 – 0.181)	0.753 (-0.149 – 0.108)	0.379 (-0.233 – 0.092)
**Right pars triangularis**	2.46 ± 0.19	2.48 ± 0.20	2.46 ± 0.19	0.162 (-0.042 – 0.245)	0.991 (-0.108 – 0.109)	0.147 (-0.257 – 0.041)
**Right pericalcarine**	1.68 ± 0.17	1.64 ± 0.14	1.69 ± 0.12	0.806 (-0.144 – 0.112)	0.696 (-0.078 – 0.116)	0.270 (-0.051 – 0.174)
**Right postcentral**	2.05 ± 0.12	2.00 ± 0.15	2.08 ± 0.08	0.924 (-0.100 – 0.091)	0.084 (-0.009 – 0.135)	0.201 (-0.037 – 0.165)
**Right posterior cingulate**	2.43 ± 0.18	2.49 ± 0.21	2.37 ± 0.18	0.446 (-0.101 – 0.226)	0.244 (-0.196 – 0.051)	0.089 (-0.336 – 0.026)
**Right precentral**	2.48 ± 0.18	2.44 ± 0.17	2.51 ± 0.16	0.385 (-0.075 – 0.192)	0.102 (-0.017 – 0.185)	0.531 (-0.108 – 0.203)
**Right precuneus**	2.31 ± 0.12	2.31 ± 0.17	2.34 ± 0.17	0.295 (-0.056 – 0.181)	0.262 (-0.039 – 0.140)	0.868 (-0.167 – 0.142)
**Right rostral anterior cingulate**	2.89 ± 0.26	2.75 ± 0.32	2.91 ± 0.25	0.542 (-0.296 – 0.158)	0.416 (-0.102 – 0.241)	0.198 (-0.085 – 0.389)
**Right rostral middle frontal**	2.36 ± 0.13	2.38 ± 0.15	2.38 ± 0.17	0.215 (-0.045 – 0.195)	0.455 (-0.057 – 0.125)	0.558 (-0.192 – 0.107)
**Right superior frontal**	2.66 ± 0.17	2.60 ± 0.17	2.67 ± 0.18	0.787 (-0.118 – 0.155)	0.466 (-0.066 – 0.141)	0.722 (-0.133 – 0.189)
**Right superior parietal**	2.14 ± 0.09	2.10 ± 0.14	2.18 ± 0.14	0.765 (-0.110 – 0.081)	0.367 (-0.039 – 0.105)	0.464 (-0.077 – 0.163)
**Right superior temporal**	2.70 ± 0.18	2.69 ± 0.22	2.70 ± 0.20	0.143 (-0.036 – 0.242)	0.427 (-0.063 – 0.147)	0.434 (-0.226 – 0.101)
**Right supramarginal**	2.50 ± 0.15	2.47 ± 0.19	2.52 ± 0.12	0.467 (-0.072 – 0.155)	0.280 (-0.039 – 0.132)	0.988 (-0.124 – 0.126)
**Right frontal pole**	2.61 ± 0.26	2.74 ± 0.28	2.65 ± 0.25	0.241 (-0.093 – 0.361)	0.761 (-0.146 – 0.198)	0.574 (-0.317 – 0.181)
**Right temporal pole**	3.50 ± 0.44	3.53 ± 0.49	3.46 ± 0.37	0.761 (-0.146 – 0.198)	0.889 (-0.297 – 0.259)	0.375 (-0.536 – 0.210)
**Right transverse temporal**	2.39 ± 0.21	2.33 ± 0.18	2.41 ± 0.20	0.410 (-0.087 – 0.209)	0.170 (-0.034 – 0.189)	0.686 (-0.129 – 0.192)
**Right insula**	3.01 ± 0.14	3.04 ± 0.25	2.98 ± 0.18	0.368 (-0.080 – 0.211)	0.418 (-0.155 – 0.065)	0.242 (-0.301 – 0.080)

Supplementary Table 3. White matter volumes (cm^3, mean ± sd)

White matter volumes (cm^3 volumes. mean ± sd)	NLp (n=26)	XXp (n=8)	XYp (n=20)	NLp vs XXp p-value (CI)	NLp vs XYp p-value (CI)	XXp vs XYp p-value (CI)
**Left bank of the superior temporal sulcus**	2.24 ± 0.51	2.43 ± 0.53	2.35 ± 0.45	0.305 (-0.212 – 0.662)	0.585 (-0.240 – 0.421)	0.598 (-0.559 – 0.330)
**Left caudal anterior cingulate**	1.79 ± 0.47	1.50 ± 0.35	1.78 ± 0.38	0.327 (-0.541 – 0.184)	0.773 (-0.235 – 0.313)	0.137 (-0.087 – 0.594)
**Left caudal middle frontal**	5.91 ± 0.96	5.40 ± 0.37	6.05 ± 1.06	0.245 (-1.333 – 0.349)	0.665 (-0.498 – 0.774)	0.170 (-0.258 – 1.368)
**Left cuneus**	2.36 ± 0.47	2.17 ± 0.27	2.25 ± 0.47	0.353 (-0.575 – 0.209)	0.315 (-0.446 – 0.147)	0.748 (-0.323 – 0.442)
**Left entorhinal**	0.55 ± 0.17	0.68 ± 0.25	0.59 ± 0.20	**0.028 (0.021 – 0.354)**	0.318 (-0.063 – 0.189)	0.158 (-0.336 – 0.058)
**Left fusiform**	5.19 ± 0.67	5.07 ± 0.67	5.36 ± 0.76	0.761 (-0.724 – 0.533)	0.471 (-0.304 – 0.647)	0.341 (-0.349 – 0.965)
**Left inferior parietal**	8.27 ± 1.16	8.81 ± 0.77	8.53 ± 1.33	0.131 (-0.245 – 1.821)	0.310 (-0.383 – 1.180)	0.405 (-1.543 – 0.648)
**Left inferior temporal**	5.50 ± 0.91	5.68 ± 1.01	5.38 ± 0.97	0.778 (-0.733 – 0.974)	0.671 (-0.783 – 0.508)	0.661 (-1.070 – 0.693)
**Left isthmus cingulate**	2.82 ± 0.61	2.69 ± 0.54	2.91 ± 0.34	0.670 (-0.321 – 0.495)	0.279 (-0.141 – 0.477)	0.272 (-0.180 – 0.608)
**Left lateral occipital**	9.17 ± 1.41	8.37 ± 1.06	8.91 ± 1.47	0.319 (-1.743 – 0.580)	0.458 (-1.205 – 0.552)	0.553 (-0.900 – 1.636)
**Left lateral orbitofrontal**	5.76 ± 0.84	5.49 ± 1.01	5.90 ± 0.95	0.598 (-1.019 – 0.593)	0.705 (-0.494 – 0.725)	0.406 (-0.509 – 1.210)
**Left lingual**	4.87 ± 0.78	4.66 ± 0.53	4.84 ± 0.83	0.828 (-0.696 – 0.560)	0.765 (-0.546 – 0.405)	0.874 (-0.620 – 0.724)
**Left medial orbitofrontal**	3.36 ± 0.65	3.37 ± 0.43	3.31 ± 0.58	0.997 (-0.534 – 0.536)	0.666 (-0.492 – 0.317)	0.818 (-0.519 – 0.414)
**Left middle temporal**	4.91 ± 0.85	4.71 ± 0.76	4.75 ± 1.05	0.548 (-1.053 – 0.567)	0.617 (-0.766 – 0.459)	0.745 (-0.713 – 0.983)
**Left parahippocampal**	1.04 ± 0.21	1.00 ± 0.21	1.11 ± 0.24	0.961 (-0.184 – 0.193)	0.287 (-0.066 – 0.218)	0.355 (-0.117 – 0.312)
**Left paracentral**	3.84 ± 0.30	3.82 ± 0.33	3.89 ± 0.60	0.881 (-0.409 – 0.352)	0.751 (-0.242 – 0.334)	0.761 (-0.400 – 0.540)
**Left cprs opercularis**	2.89 ± 0.39	2.60 ± 0.38	2.75 ± 0.57	0.300 (-0.617 – 0.194)	0.436 (-0.427 – 0.187)	0.657 (-0.387 – 0.600)
**Left pars orbitalis**	0.97 ± 0.18	0.99 ± 0.18	1.03 ± 0.20	0.821 (-0.147 – 0.185)	0.332 (-0.065 – 0.187)	0.395 (-0.102 – 0.249)
**Left pars triangularis**	3.06 ± 0.46	2.78 ± 0.27	2.90 ± 0.55	0.400 (-0.598 – 0.243)	0.520 (-0.421 – 0.216)	0.694 (-0.378 – 0.557)
**Left pericalcarine**	2.83 ± 0.61	2.98 ± 0.48	2.91 ± 0.60	0.524 (-0.357 – 0.692)	0.819 (-0.352 – 0.442)	0.719 (-0.620 – 0.434)
**Left postcentral**	6.60 ± 0.87	6.36 ± 0.73	6.87 ± 1.15	0.520 (-1.118 – 0.574)	0.561 (-0.454 – 0.826)	0.351 (-0.519 – 1.400)
**Left posterior cingulate**	2.98 ± 0.67	2.73 ± 0.53	3.09 ± 0.49	0.827 (-0.560 – 0.450)	0.320 (-0.191 – 0.573)	0.097 (-0.079 – 0.879)
**Left precentral**	12.03 ± 1.38	11.60 ± 1.29	12.71 ± 1.58	0.391 (-1.831 – 0.730)	0.184 (-0.320 – 1.618)	0.104 (-0.254 – 2.539)

**Left precuneus**	7.71 ± 1.08	7.59 ± 0.91	8.01 ± 1.29	0.801 (-1.138 – 0.883)	0.557 (-0.540 – 0.989)	0.544 (-0.722 – 1.330)
**Left rostral anterior cingulate**	2.03 ± 0.34	1.78 ± 0.53	2.04 ± 0.54	0.170 (-0.671 – 0.122)	0.906 (-0.318 – 0.282)	0.303 (-0.243 – 0.746)
**Left rostral middle frontal**	11.08 ± 1.76	10.03 ± 1.59	10.99 ± 1.98	0.119 (-2.880 – 0.341)	0.636 (-1.507 – 0.930)	0.240 (-0.660 – 2.493)
**Left superior frontal**	16.11 ± 2.32	15.27 ± 1.94	16.27 ± 2.50	0.404 (-2.969 – 1.218)	0.929 (-1.513 – 1.655)	0.335 (-1.065 – 2.988)
**Left superior parietal**	9.79 ± 0.94	10.49 ± 1.07	10.42 ± 1.43	0.146 (-0.266 – 1.730)	0.173 (-0.236 – 1.274)	0.699 (-1.253 – 0.856)
**Left superior temporal**	7.02 ± 0.88	6.77 ± 1.04	7.21 ± 1.00	0.590 (-1.083 – 0.623)	0.563 (-0.458 – 0.832)	0.292 (-0.456 – 1.444)
**Left supramarginal**	7.44 ± 1.34	7.06 ± 1.27	7.44 ± 1.08	0.589 (-1.384 – 0.796)	0.788 (-0.714 – 0.935)	0.522 (-0.746 – 1.426)
**Left frontal pole**	0.32 ± 0.06	0.31 ± 0.04	0.31 ± 0.05	0.557 (-0.063 – 0.035)	0.526 (-0.049 – 0.025)	0.870 (-0.039 – 0.046)
**Left temporal pole**	0.56 ± 0.11	0.65 ± 0.11	0.61 ± 0.15	0.186 (-0.036 – 0.180)	0.204 (-0.030 – 0.134)	0.645 (-0.163 – 0.103)
**Left transverse temporal**	0.92 ± 0.20	0.89 ± 0.11	0.82 ± 0.13	0.691 (-0.177 – 0.118)	0.146 (-0.194 – 0.030)	0.317 (-0.177 – 0.060)
**Left insula**	7.09 ± 0.79	6.84 ± 1.03	7.38 ± 1.09	0.633 (-1.038 – 0.638)	0.355 (-0.340 – 0.928)	0.292 (-0.487 – 1.541)
**Right bank of the superior temporal sulcus**	2.33 ± 0.43	2.34 ± 0.23	2.28 ± 0.32	0.994 (-0.322 – 0.325)	0.554 (-0.317 – 0.172)	0.513 (-0.315 – 0.162)
**Right caudal anterior cingulate**	1.87 ± 0.47	1.59 ± 0.32	1.84 ± 0.32	0.247 (-0.545 – 0.144)	0.849 (-0.285 – 0.236)	0.125 (-0.071 – 0.539)
**Right caudal middle frontal**	5.37 ± 0.78	4.99 ± 1.04	5.46 ± 0.84	0.299 (-1.131 – 0.356)	0.725 (-0.464 – 0.661)	0.256 (-0.358 – 1.274)
**Right cuneus**	2.54 ± 0.55	2.32 ± 0.36	2.41 ± 0.41	0.273 (-0.664 – 0.192)	0.310 (-0.489 – 0.159)	0.503 (-0.260 – 0.513)
**Right entorhinal**	0.52 ± 0.14	0.58 ± 0.18	0.51 ± 0.16	0.337 (-0.070 – 0.200)	0.871 (-0.110 – 0.094)	0.410 (-0.225 – 0.096)
**Right fusiform**	4.93 ± 0.80	4.90 ± 1.09	5.01 ± 0.82	0.926 (-0.808 – 0.736)	0.804 (-0.511 – 0.656)	0.578 (-0.588 – 1.027)
**Right inferior parietal**	9.50 ± 1.38	9.38 ± 1.15	9.86 ± 1.64	0.819 (-1.118 – 1.406)	0.329 (-0.487 – 1.423)	0.638 (-1.094 – 1.746)
**Right inferior temporal**	5.07 ± 0.69	4.94 ± 0.81	5.10 ± 0.84	0.703 (-0.819 – 0.557)	0.938 (-0.500 – 0.541)	0.609 (-0.546 – 0.910)
**Right isthmus cingulate**	2.53 ± 0.57	2.29 ± 0.44	2.61 ± 0.32	0.879 (-0.389 – 0.334)	0.246 (-0.114 – 0.433)	0.096 (-0.052 – 0.591)
**Right lateral occipital**	9.29 ± 1.55	8.54 ± 0.81	8.87 ± 1.09	0.410 (-1.581 – 0.657)	0.468 (-1.154 – 0.539)	0.700 (-0.754 – 1.103)
**Right lateral orbitofrontal**	6.00 ± 0.92	5.89 ± 1.03	6.04 ± 1.02	0.837 (-0.955 – 0.777)	0.967 (-0.669 – 0.642)	0.779 (-0.792 – 1.044)
**Right lingual**	5.04 ± 0.86	4.85 ± 0.78	5.12 ± 1.14	0.703 (-1.005 – 0.683)	0.996 (-0.640 – 0.637)	0.786 (-0.782 – 1.021)
**Right medial orbitofrontal**	3.64 ± 0.67	3.50 ± 0.48	3.50 ± 0.48	0.473 (-0.708 – 0.334)	0.368 (-0.572 – 0.216)	0.873 (-0.388 – 0.454)
**Right middle temporal**	5.62 ± 0.92	5.33 ± 0.64	5.51 ± 0.84	0.484 (-1.036 – 0.499)	0.848 (-0.636 – 0.525)	0.409 (-0.405 – 0.958)
**Right parahippocampal**	1.03 ± 0.18	1.02 ± 0.17	1.03 ± 0.21	0.800 (-0.190 – 0.148)	0.785 (-0.145 – 0.110)	0.939 (-0.163 – 0.152)

**Right paracentral**	4.62 ± 0.55	4.50 ± 0.35	4.88 ± 0.75	0.641 (-0.648 – 0.403)	0.360 (-0.215 – 0.580)	0.272 (-0.243 – 0.817)
**Right cprs opercularis**	2.62 ± 0.35	2.35 ± 0.25	2.60 ± 0.37	0.179 (-0.497 – 0.095)	0.938 (-0.215 – 0.233)	0.172 (-0.102 – 0.535
**Right pars orbitalis**	1.22 ± 0.21	1.17 ± 0.24	1.27 ± 0.22	0.758 (-0.226 – 0.165)	0.342 (-0.077 – 0.218)	0.232 (-0.085 – 0.331)
**Right pars triangularis**	3.03 ± 0.41	2.86 ± 0.48	3.07 ± 0.51	0.335 (-0.601 – 0.209)	0.964 (-0.299 – 0.313)	0.264 (-0.199 – 0.691)
**Right pericalcarine**	2.85 ± 0.64	2.90 ± 0.68	2.89 ± 0.78	0.789 (-0.534 – 0.698)	0.986 (-0.470 – 0.462)	0.759 (-0.781 – 0.579)
**Right postcentral**	6.66 ± 0.86	6.69 ± 0.85	6.77 ± 0.95	0.772 (-0.910 – 0.680)	0.996 (-0.600 – 0.603)	0.737 (-0.681 – 0.947)
**Right posterior cingulate**	2.88 ± 0.55	2.70 ± 0.45	2.86 ± 0.47	0.908 (-0.461 – 0.411)	0.791 (-0.286 – 0.373)	0.478 (-0.285 – 0.588)
**Right precentral**	12.17 ± 1.21	12.06 ± 1.03	12.63 ± 2.11	0.750 (-1.610 – 1.168)	0.549 (-0.736 – 1.366)	0.629 (-1.254 – 2.028)
**Right precuneus**	7.88 ± 0.91	7.75 ± 1.20	8.00 ± 1.29	0.952 (-1.008 – 0.949)	0.707 (-0.601 – 0.879)	0.789 (-1.034 – 1.345)
**Right rostral anterior cingulate**	1.39 ± 0.30	1.32 ± 0.26	1.48 ± 0.40	0.793 (-0.325 – 0.250)	0.536 (-0.150 – 0.285)	0.488 (-0.226 – 0.459)
**Right rostral middle frontal**	11.27 ± 2.02	10.40 ± 2.10	11.42 ± 1.52	0.192 (-2.765 – 0.570)	0.962 (-1.232 – 1.291)	0.130 (-0.363 – 2.642)
**Right superior frontal**	15.80 ± 2.28	14.73 ± 2.06	16.08 ± 2.81	0.431 (-2.999 – 1.302)	0.695 (-1.308 – 1.945)	0.294 (-1.073 – 3.379)
**Right superior parietal**	9.72 ± 1.14	9.70 ± 1.30	9.93 ± 1.36	0.926 (-1.047 – 1.148)	0.708 (-0.675 – 0.986)	0.806 (-1.048 – 1.332)
**Right superior temporal**	5.98 ± 0.77	5.88 ± 0.80	5.93 ± 1.12	0.585 (-1.038 – 0.592)	0.674 (-0.746 – 0.487)	0.858 (-0.883 – 1.052)
**Right supramarginal**	7.08 ± 1.03	7.23 ± 0.77	7.40 ± 1.18	0.709 (-0.764 – 1.114)	0.438 (-0.434 – 0.986)	0.841 (-0.897 – 1.091)
**Right frontal pole**	0.39 ± 0.06	0.39 ± 0.05	0.40 ± 0.06	0.981 (-0.050 – 0.049)	0.849 (-0.0340 – 0.041)	0.969 (-0.051 – 0.050)
**Right temporal pole**	0.56 ± 0.11	0.58 ± 0.19	0.56 ± 0.12	0.791 (-0.122 – 0.093)	0.753 (-0.094 – 0.068)	0.788 (-0.114 – 0.148)
**Right transverse temporal**	0.57 ± 0.13	0.62 ± 0.08	0.67 ± 0.23	0.609 (-0.110 – 0.186)	0.104 (-0.020 – 0.204)	0.598 (-0.133 – 0.225)
**Right insula**	6.85 ± 0.84	6.52 ± 1.08	7.19 ± 0.98	0.632 (-1.030 – 0.632)	0.196 (-0.219 – 1.038)	0.173 (-0.311 – 1.624)
